# Novel porphyrin derivatives as corrosion inhibitors for stainless steel 304 in acidic environment: synthesis, electrochemical and quantum calculation studies

**DOI:** 10.1038/s41598-023-44873-2

**Published:** 2023-10-16

**Authors:** A. S. Fouda, H. M. Abdel-Wahed, M. F. Atia, A. El-Hossiany

**Affiliations:** 1 Department of Chemistry, Faculty of Science, Mansoura University, Mansoura, 35516 Egypt; 2 Department of Chemistry, Faculty of Science, Tanta University, Tanta, Egypt; 3 Delta Fertilizers Company on Talkha, Talkha, Egypt

**Keywords:** Electrochemistry, Materials science

## Abstract

A Novel 5,10,15,20-tetra (thiophen-2-yl) porphyrin **(P1)** and 5,10,15,20-tetrakis (5-Bromothiophen-2-yl) porphyrin **(P2)** were successfully synthesized, and their chemical structures were proved based on its correct elemental analysis and spectral data (IR and ^1^H-NMR). These compounds were examined as corrosion inhibitors for stainless steel 304 (SS304) in 2 M HCl utilizing mass reduction (MR) and electrochemical tests at inhibitor concentration (1 × 10^–6^–21 × 10^–6^ M). The protection efficiency (IE %) was effectively enhanced with improving the concentration of investigated compounds and reached 92.5%, 88.5% at 21 × 10^–6^ M for P1 & P2, respectively and decreases with raising the temperature. Langmuir's isotherm was constrained as the best fitted isotherm depicts the physical–chemical adsorption capabilities of P1 & P2 on SS304 surface with change in ΔG^o^_ads_ = 22.5 kJ mol^−1^. According to the PDP data reported, P1 and P2 work as mixed find inhibitors to suppress both cathodic and anodic processes. Porphyrin derivatives (P1 & P2) are included on the surface of SS304, according to surface morphology techniques SEM/EDX and AFM. Quantum calculations (DFT) and Monte Carlo simulation (MC) showed the impact of the chemical structure of porphyrin derivatives on their IE %.

## Introduction

Stainless steel is crucial to both daily life and the engineering industry. Having outstanding mechanical and thermal properties and being one of the most often utilized structural materials. Identification of stainless steel corrosion is crucial since it not only reduces the material’s quality but also results in significant financial losses^1^. The use of corrosion inhibitors in pickling baths is one of their principal functional uses. Pickling baths are used to clean metal parts by removing contaminants such as oil, impurities, oxide layers, and corrosion products from the surface of the material. Hydrochloric acid, sulfuric acid, phosphoric acid, hydrofluoric acid, and organic acids including citric acid, acetic acid, and oxalic acid can all be used alone or in combination for this technique. One of the most used anticorrosion methods, the inclusion of organic inhibitors, significantly lowers electrode reactivity^2, 3^. Steel is regarded as the most useful material because of its many manufacturing applications and great mechanical properties. Numerous operations employ the acidic solution, such as metal picking to reduce scale, petrochemical industries using chemical processes, and the manufacture of rocket tool components^4^. Corrosion control techniques include the choice and use of corrosion inhibitors. The most beneficial, cost-effective and feasible strategy is outperformed by the employment of chemical inhibitors, Corrosion inhibitors may be categorized into two main groups: organic and inorganic chemicals. Due to their potential for toxicity and pollution, inorganic corrosion inhibitors are regulated, whereas organic inhibitors are the most widely used method of preventing metal corrosion in hostile settings^5–8^. According to established research, ultimate organic inhibitors function by adsorbing on the metal surface. The heteroatoms phosphorus, oxygen, nitrogen, and sulphur, as well as triple bonds and aromatic rings, are all targets for the binding of inhibitors. These compounds are absorbed on the metallic surface, blocking the active corrosion sites. The bulk of these chemicals, however, are pricy and environmentally harmful^9–13^. Organic molecules with heteroatoms had an affinity for the metal's surface, and they could be adsorbed by blocking the active sites and forming a thin layer, which would stop corrosive chemicals from passing through the metal^14–16^. Because they can improve effective adsorption by forming covalent bonds with metal atoms' vacant d-orbitals, organic ligands with π-bonds, C=N, and lone electron pairs (S, O, and N) would create strong inhibitive properties^17–19^. The S and N atoms have been shown to have the capability to form stable complexes that are closely arranged in the coordination sphere of metal ions^20^. Numerous of these substances act as powerful ligands and coordinate bonds with transition metals. The creation of a protective coating that protects the metals from aggressive surroundings is caused by the adsorption of these substances onto metal substrates^21–25^.

The protective properties of some macrocyclic composites namely: (PTAT); (PTAB); (POAB); (OAH), (BOAH), (MOAT), (BMOAT), (DBOAD) and (TBOAD) were studied by Quraishi and Rawat on the mild steel corrosion in acidic environment^26^. Singh et al.^27^ investigated the effects of “(THP) 4,4′,4′′′,4′′′′-(porphyrin-5,10,15,20-tetrayl)tetrakis(benzoic acid), (HPTB) 5,10,15,20-tetrakis(4-hydroxyphenyl)-21*H*,23*H*-porphyrin, (T4PP) 5,10,15,20-tetra(4-pyridyl)-21*H*,23*H*-porphyrin and (TPP) 5,10,15,20-tetraphenyl-21*H*,23*H*-porphyrin on the dissolution of N80 steel in 3.5% NaCl environment saturated with CO_2_”. The results showed that all the macroheterocyclic composites are efficient N80 steel corrosion inhibitors, with degrees of protection ranging from 85 to 91%^28^, but for J55 steel. At a dosage of 0.4 mM, it was revealed that PF-2 offers about 93% more protection than PF-1. Mild steel dissolution in 5% H_2_SO_4_ was tested using (TPyP) 5, 10, 15, 20-tetrakis (4-pyridyl)-21H, 23H-porphin as an inhibitor^29^. “The findings demonstrated that TPyP inhibits the corrosion of steel by more than 50% and that this inhibitory action is temperature and concentration dependent. The current density in the active region of potentials is likewise markedly reduced when hydrophenyl is used in place of pyridyl”^30^. The same authors^31^ additionally coated 5, 10, 15, 20–20-tetra (4-methylphenyl)-21H, 23H- porphyrin with polyaniline to generate a protective layer. It turns out that in 0.1 M H_2_SO_4_, this treatment protects mild steel by 80%. In 3.5% NaCl saturated with CO_2_, several porphin derivatives were employed as corrosion inhibitors for J55^32^ and N80 steels^33^. According to studies, the protective effect improves with porphyrin content but decreases with temperature. The reasons for preparation and the use these porphyrin compounds as corrosion inhibitors are due to: easily prepared, have high molecular size, porphyrin and their derivatives are assumed to form strong chelating complexes with metallic atoms (due to the presence of four nitrogen atoms with free unshared electron pairs and various aromatic rings with extensively conjugated π-electrons).

The purpose of the current investigation was to prepare newly generated macrocyclic organic compounds (porphyrin P1 & P2) (Table 1) and to examine their inhibition efficacy for SS304 in 2 M HCl using various electrochemical processes and MR at different inhibitor's concentration. Additionally, to examine how they adhered to the surface of SS304. DFT was used to connect the experimental findings to the quantum chemical properties of the generated inhibitors^34^.

**Table 1:** Chemical structure of the porphyrin derivatives (P1 & P2).

P1	P2
	
[5,10,15,20-tetra(thiophen-2-yl)porphyrin]	5,10,15,20-tetrakis(5-bromothiophen-2-yl)porphyrin
Mol. Formula: C_36_H_22_N_4_S_4_	C_36_H_18_Br_4_N_4_S_4_
MW = 638.84 g mol^−1^	MW = 954.42 g mol^−1^

## Experiment

### Synthesis of porphyrin derivatives

They were synthesized allowing to earlier reported work^35–38^.

### Synthesis of 5, 10, 15, 20-tetra (thiophen-2-yl) porphyrin (P1)

**Figure 1:**
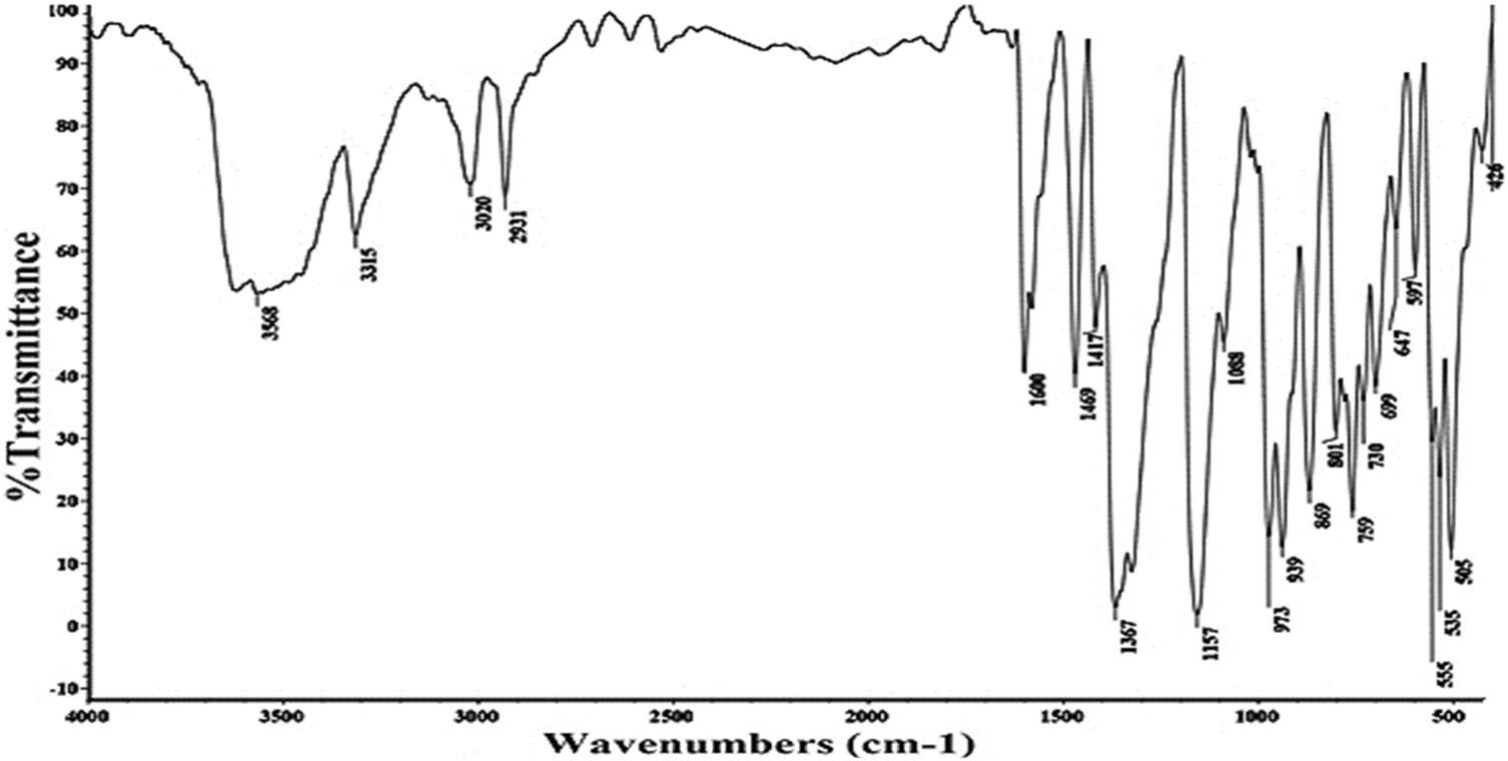
IR spectrum of compound **P1.**

Yield: “(70%); m.p. < 300 °C; IR (KBr) *v*/cm^−1^: 3315 (NH), 3020 (CH-stretching), 1600 (C=C) Fig. 1; ^1^H-NMR (DMSO-*d*_*6*_) (Fig. S1): δ (ppm): 6.24 (d, 2H, pyrrolic-CH), 6.31 (d, 2H, pyrrolic-CH), 6.40 (d, 2H, pyrrolic-CH), 7.00–7.32 (m, 8H, thiophene-H), 7.84 (d, 2H, pyrrolic-CH), 8.87 (s, 1H, NH), 8.88, 8.93 (m, 4H, thiophene-H), 9.64 (s, 1H, NH); ^13^C-NMR (DMSO-*d*_*6*_) (Fig. S2): δ (ppm): 115.0, 119.1, 120.2, 127.8, 128.1, 130.6, 135.1, 136.5, 140.1, 141.2, 142.5, 158.9, 164.5; UV (DMF) (Fig. S3): 426 nm. Anal. Calcd. for C_36_H_22_N_4_S_4_ (638.84): C, 67.68; H, 3.47; N, 8.77%. Found: C, 67.59, H, 3.40; N, 8.71%”.

**Figure 2:**
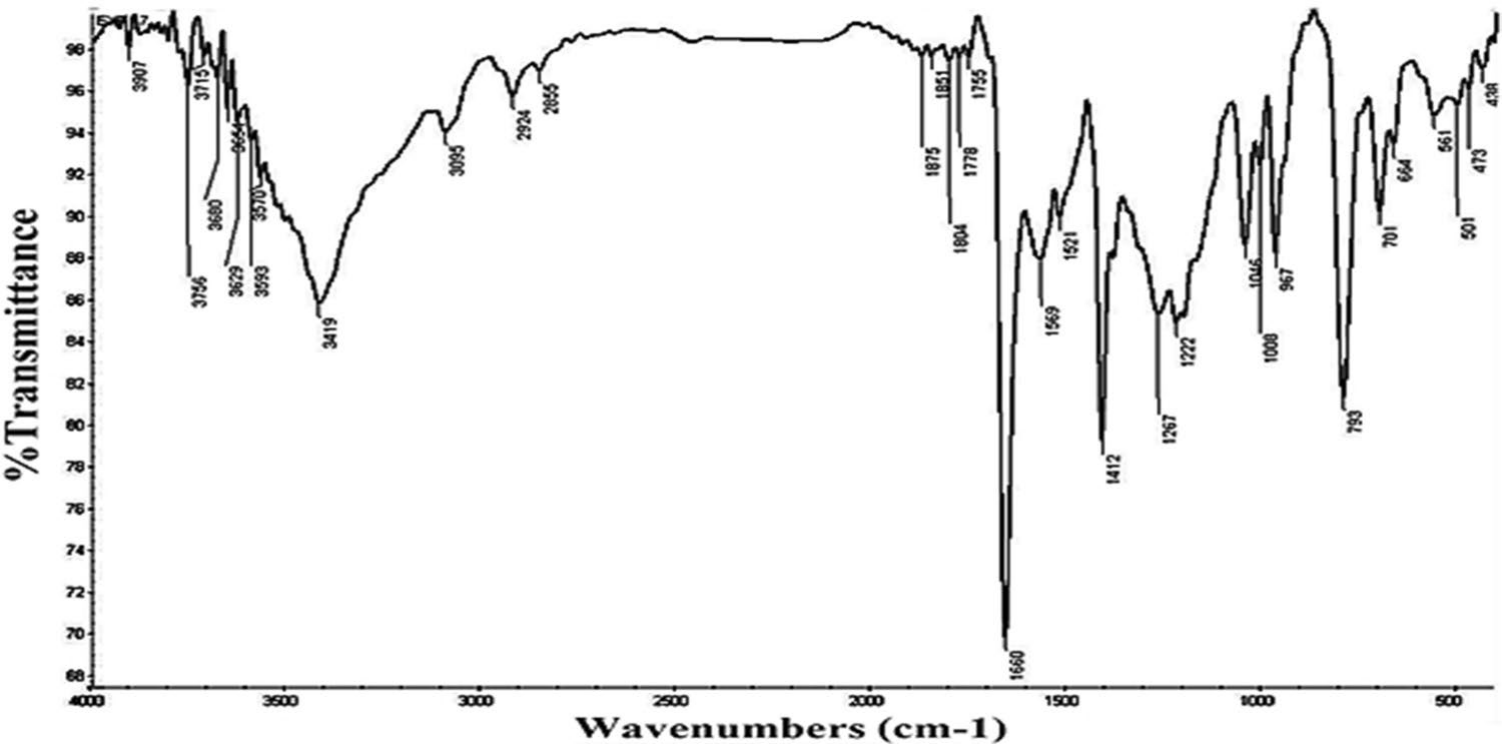
IR spectrum of compound **P2.**

### Synthesis of 5, 10, 15, 20-tetrakis (5-bromothiophen-2-yl) porphyrin (P2)

Yield: “(76%); m.p. < 300 °C; IR (KBr) *v*/cm^−1^: 3419 (NH) Fig. 2, 793 (C–Br); ^1^H-NMR (Fig. S4) (DMSO-*d*_*6*_) (SF4): δ (ppm): 6.24 (d, 2H, pyrrolic-CH), 6.38 (d, 2H, pyrrolic-CH), 6.45 (d, 2H, pyrrolic-CH), 6.88 (d, 4H, thiophene-H), 7.05 (d, 4H, thiophene-H), 7.84 (d, 2H, pyrrolic-CH), 8.78 (s, 1H, NH), 9.83 (s, 1H, NH); ^13^C-NMR (DMSO-*d*_*6*_) (Fig. S5): δ (ppm): 116.1, 117.8, 118.2, 118.8, 125.5, 133.4, 135.2, 137.7, 138.3, 139.9, 140.9, 142.0, 157.2, 166.8; UV (DMF) (Fig. S6): 430 nm. Anal. Calcd. for C_36_H_18_Br_4_N_4_S_4_ (954.42): C, 45.30; H, 1.90; N, 5.87%. Found: C, 45.25, H, 1.83; N, 5.82%”.

### Composition of SS304 samples

The chemical make-up of the SS304 utilized in this study includes (0.08% C, 0.045% P, 2% Mn, 0.75% Si, 0.03% S, 20% Cr, 8% Ni, 0.1% Al and Fe remainder), which is exactly similar to the ASTM SS304 standard. All specimens were cold cut from stainless steel 304 plates with a surface area of 1 cm^2^ for the working electrodes in electrochemical measurements and surface examination. The mass reduction approach was applied using coins that were 2 × 2 × 0.2 cm in size. Each sample was prepared in the laboratory by being scrubbed with numerous abrasive sandpapers ranged (180–2000) then it was soaked in acetone as a degreasing solution thus washed with double-distilled water and dried between two filter papers resulting in mirror-like finish.

### Chemicals

A 37% HCl corrosive environment was used (AR grade). The bidistilled water was used to prepare the appropriate dosages of acid. To acquire different inhibitor concentrations, a (1 × 10^–3^ M) stock solution of porphyrin organic inhibitors was diluted with bidistilled water (1–21 × 10^–6^ M). The maximum dosage of porphyrin molecule in 2 M HCl was estimated to be 21 × 10^–6^ M. The porphyrin compounds utilized in this study have higher molecular weights, are non-toxic, highly soluble in water, and include a sizable amount of donating atoms (N and S). Here is a list of the structures that they utilize:

### Chemical test: Mass reduction (MR) test

Using a water thermostat and 100 ml of 2 M HCl solution, test coins made of SS304 were subjected to various concentrations of the porphyrin composites under research between 298 and 318 K. After the proper amount of dipping time, the coins were removed and weighed. At a particular time (180 min), the average MR for the tested samples was determined in mg cm^−2^.

### Electrochemical tests

The electrochemical experiments were done in glass reaction reactor involving three electrodes. A working electrode fabricated from SS304 (1 cm^2^) and prepared in same manner as in weight loss method, a counter electrode made up from platinum foil (1 cm^2^) and standard calomel electrode via Luggin capillary. All three were embedded in epoxy resins to expose the desired unified geometrical surface area then dipped in freshly prepared test solution at room temperature and stabilized for 30 min before each experiment until reaching steady state under unstirred condition, where the potentials are displayed versus normalized hydrogen electrode. The electrode was constructed of SS304. Through 250 mV_SCE_, the potential was automatically altered. For PDP, it involves sweeping the potential in positive direction until 100 mV and then reverses the direction toward more negative until − 100 mV at scan rate 0.2 mVs^−1^. For electrochemical impedance spectroscopy, we recorded results at frequency (1 × 10^5^–0.1 Hz) and amplitude of 10 mV, Electrochemical measurements were carried out using Gamry instruments (Series G 750™—Potentiostat/Galvanostat/ZRA device)) then the graphing, fitting and recording were done using Software called Echem Analyst 5.5. All results were obtained and the process was repeated three times to ensure the validity results.

### Surface examination techniques

Analyzing the SS304 surface is crucial to identify the morphology, proving the adsorption of porphyrin derivatives (P1 & P2) and assessment of their impact as inhibitors. Our specimens were prepared by grounding the SS304 coupons to a grit of 4000 and then polished with a number of sand-papers. The prepared metal sheets were immersed in 2 M HCl solution for 24 h at 298 K without the addition of the inhibitors to evaluate the influence of corrosive medium on metal morphology. Analogous actions were conducted but with 21 × 10^–6^ M of inhibitor solutions. A comparison between the morphologies of samples attacked by the corrosive medium and those of the inhibited ones. These investigations were fulfilled by AFM (Model. FlexAFM3), SEM model A Jeol JSM-5400 instrument was used in the investigation.

### Computational methods

#### Quantum chemical calculations

Using Material Studio version 7.0 semi-empirical approaches using the density functional theory (DFT), the entire quantum chemistry study has been conducted. Semi-empirical methodology was used to calculate molecular orbitals. The molecules were optimized by choosing B3LYB (Becke-3-parameters-lee-yang-parr) with DNP functions while setting the fine quality. Fine convergence and global orbital cutoffs were utilized as well as setting water as solvent which impact the treatment via COSMO controls.

#### Monte-Carlo simulations (MC)

Using MC, the optimal positioning of P1 & P2 inhibitors on the apparent of Fe (1 1 0) was evaluated. According to the literature^39^, it is believed that the Fe (1 1 0) crystal surface is used in this simulation due to its most stable. In order to simulate the solvent action during the corrosion process, 100 water molecules were employed to examine the adsorption of uncharged and protonated inhibitor molecules. The estimation module was initially used to carry out the geometrical optimization of water and the inhibitor molecule. Compass stimulation along with force field were implemented to porphyrin derivatives (P1 & P2) on Fe (1 1 0) optimized surface. The substrate-adsorbate system configuration space was searched using the Monte-Carlo approach to identify low-energy adsorption sites where the temperature gradually decreases.

## Results and discussion

### Chemistry of porphyrin derivatives

**Figure 3:**
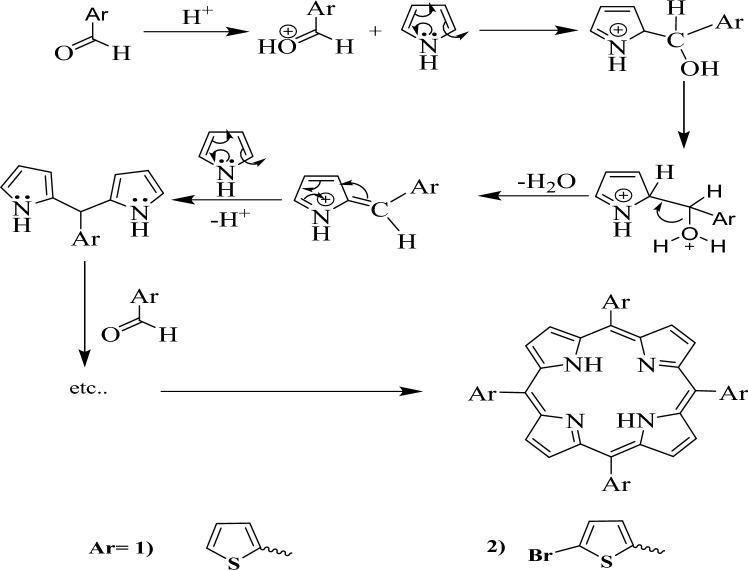
General route for the synthesis of porphyrin derivatives.

There has been an important attention in porphyrin derivatives^40^. Therefore, it was interesting to the synthesis of [porphyrin] (P1) and (P2) according to the following Fig. 3.

The ^1^HNMR of compound **P1** displayed two singlet signals at δ 8.87 and 9.64 for 2NH, and multiplet signal at 7.00–7.32 ppm for aromatic protons (SF2). Additionally, the ^1^HNMR spectrum of composite **P2** showed two singlet signals at δ 8.78 and 9.83 ppm for 2NH (SF6).

### Mass reduction (MR) test

**Figure 4:**
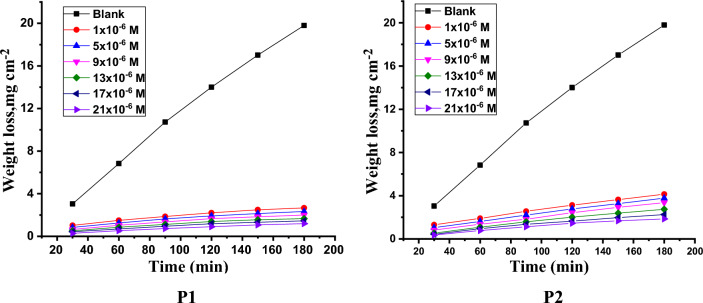
Time-MR bends of SS304 in 2 M HCl using and devoid of various doses of porphyrin derivatives (P1 & P2) at 25 °C.

Measurements using MR have several practical uses^41^. It is the first technique for determining how corrosive an environment is to a particular substance. The main benefits of this approach are its availability, applicability in all settings, and ease of calculation of the corrosion rate. Specimens were cleaned in accordance with ASTM standard G1-3^42^ before to the start of any experiment. Tests gotten at altered time intervals lacking and existence 1–21 × 10^–6^ M of the porphyrin derivatives (P1 & P2) on SS304 pieces were done. ΔW is given from the Eq (1):

1 $$\Delta W = \frac{{W_{1} - W_{2} }}{a}$$

where, W_1_ and W_2_ are the mass of specimens previously and later reaction, correspondingly and the surface area in cm^2^. IE % was calculated from the Eq. (2):

2 $$IE \% = \frac{{\Delta W - \Delta W_{i} }}{\Delta W} \times 100$$

where ΔW and ΔWi are the mass reduced/a without and existence the porphyrin derivatives (P1 & P2), individually. Figure 4 display the calculated mass reduction for SS304 at 25 ± 1 °C existence and absence altered doses ranging from 1 × 10^–6^ M to 21 × 10^–6^ M for porphyrin derivatives (P1 & P2).

The final data displayed that IE % of the investigated compounds reduced with increasing temperature Table 2 and increased with improving inhibitor concentration.

**Table 2:** Impact of temperature on the IE % of porphyrin derivatives (P1 & P2) at 120 min in 2 M HCl solution.

Inhibitor	Conc., × 10^6^ M	IE %				
		25 °C	30 °C	35 °C	40 °C	45 °C
**P1**	1	84.3	82.8	79.4	78.4	76.9
	5	86.3	84.1	82.0	80.2	79.4
	9	88.1	87.9	84.6	81.7	80.1
	13	90.1	88.8	87.3	83.3	81.8
	17	91.6	89.9	88.9	86.6	83.7
	21	93.6	91.2	90.8	88.4	86.2
**P2**	1	77.7	74.0	70.0	64.5	60.9
	5	80.2	77.6	72.8	68.2	63.8
	9	82.7	80.4	75.2	71.0	66.7
	13	85.6	82.7	78.1	74.2	70.3
	17	88.3	85.9	81.3	77.0	74.0
	21	89.6	87.7	85.1	80.6	77.8

### Effect of temperature on the corrosion process

#### Kinetic-thermodynamic corrosion parameters

**Figure 5:**
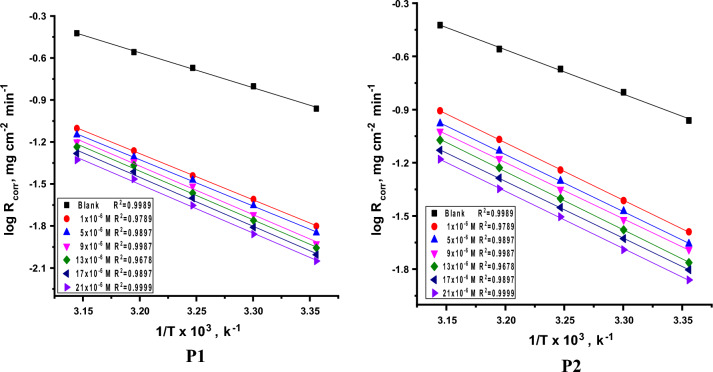
Log R_corr_ vs. 1/T of compounds (P1 & P2) and free sample at temperature range 25–45 °C.

Analysis of the impact of temperature on the corrosion of SS304 in terms of activation energy was done using the Arrhenius equation. Calculating the standard activation energy E^*^_a_ was done using the MR results. Arrhenius diagrams of SS304 in 2 M HCl solutions in the absence and presence of porphyrin derivatives (P1 & P2) at various concentrations and temperatures ranging from 298 to 318 K are shown in Table 3. The slope of the line drawn by graphing 1000/T (Fig. 5) in accordance with log k_corr_ was used to determine the activation energy (E^*^_a_) value in accordance with the Arrhenius formula Eq. 3^43^:

3 $$R_{corr} = A\exp \left( {\frac{{ - E_{a}^{*} }}{RT}} \right)$$

**Table 3:** The R_corr_ of the investigated porphyrin derivatives (P1 & P2) and the free sample at 120 min dipping.

Inhibitor	Conc., × 10^6^ M	25 °C	30 °C	35 °C	40 °C	45 °C	
**P1**	Blank	0.1167	0.1583	0.2143	0.2848		0.3025
	1	0.0184	0.0272	0.0441	0.0607		0.0700
	5	0.0160	0.0251	0.0385	0.0581		0.0624
	9	0.0139	0.0191	0.0331	0.0515		0.0601
	13	0.0116	0.0177	0.0273	0.0471		0.0552
	17	0.0099	0.0160	0.0238	0.0378		0.0493
	21	0.0075	0.0139	0.0196	0.0327		0.0417
**P2**	1	0.0260	0.0411	0.0660	0.1017		0.1183
	5	0.0231	0.0355	0.0598	0.0907		0.1094
	9	0.0202	0.0310	0.0545	0.0826		0.1007
	13	0.0168	0.0274	0.0481	0.0733		0.0800
	17	0.0136	0.0222	0.0410	0.0654		0.0785
	21	0.0121	0.0195	0.0327	0.0553		0.0670

Since “k_corr_” is rate of SS304 corrosion, “R and T” are, respectively, gas constant and Kelvin temperature. At 21 × 10^–6^ M, the activation energy of the SS304 in inhibited solution is 72, 70 kJ mol^−1^ for P1 and P2, respectively. 47.9 kJ mol^−1^ is the Figure for the uninhibited solution. The inhibitor solution’s high activation energy results from the protective layer they create, which lowers the energy barrier for charge and mass transfer and prevents metal dissolution. Hence, the inhibitory system's dissolution is a slow process^44^.

**Figure 6:**
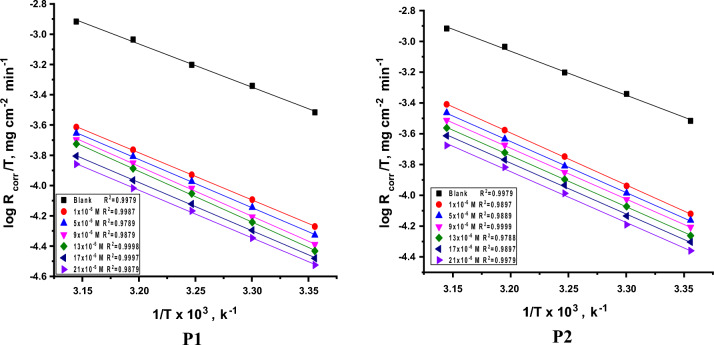
Log (R_corr_/T) vs 1/T of compounds (P1 & P2) and free sample at temperature range 25–45 °C.

The transition-state equation (Fig. 6) was used to attain the enthalpy (ΔH^*^) and entropy (ΔS^*^) data of activation that it was as follows (Eq. 4):

4 $$R_{corr} = \frac{RT}{{Nh}} exp\left( {\frac{{\Delta S^{*} }}{R}} \right) exp\left( {\frac{{ - \Delta H^{*} }}{RT}} \right)$$

Table 4 illustrates the measured activation parameters. According to the current study, E^*^_a_ values are larger with the examined inhibitors present than they are when the 2 mol L^−1^ HCl solution is used alone, suggesting that this behavior may be attributable to physical adsorption into the SS304 surface. The exothermic nature is shown by the negative sign of (∆H^*^)”. This means that the adsorption may be chemical or physical. Also, the rise in the values of ΔS^*^ (lower negative values) in the presence of the examined derivatives as compared to free acid solution indicated an increase in the order that happened when switching from the reagents to the steel/solution interface^45^.

**Table 4:** Activation data of the liquefaction of SS304 in 2 M HCl with and without of porphyrin compounds (P1&P2) at 25–45 °C.

Inhibitor	Conc., × 10^6^ M	Activation parameters		
		E_a_*	ΔH*	− ΔS*
		kJ mol^−1^	kJ mol^−1^	J mol^−1^ K^−1^
Blank	–	47.9	45.3	120.7
**P1**	1	67.4	64.8	72.3
	5	68.8	66.2	68.5
	9	69.7	67.2	65.7
	13	70.5	67.9	64.0
	17	71.3	68.7	62.7
	21	72.0	69.4	62.1
**P2**	1	65.3	62.7	70.1
	5	67.5	64.9	64.5
	9	68.1	65.5	63.5
	13	68.8	66.2	62.3
	17	69.1	66.5	61.5
	21	70.0	67.4	60.2

#### Adsorption isotherm behavior

**Figure 7:**
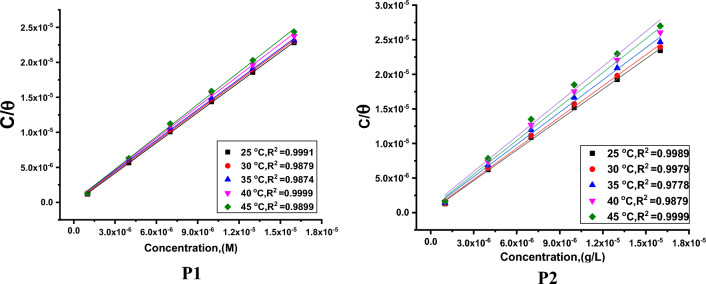
Langmuir bends of P**1** & P2 on SS304 surface in 2 M HCl at various temperatures.

Different adsorption isotherms are fitted graphically to find out that adsorption obeys Langmuir adsorption isotherm (Fig. 7) with correlation coefficient (R^2^) near to 1. Applying Langmuir equation (Eq. 5) reflects the correlation between surface coverage (ϴ) and the inhibitors equilibrium concentration (C) in the bulk solution and assesses adsorption equilibrium constant (K_ads_)^46, 47^.

5 $$\frac{{C_{inh} }}{\theta } = \frac{1}{{K_{ads} }} + C_{inh}$$

Then we calculated standard Gibbs free energy (ΔG_ads_°) from Eq. (6):

6 $$K_{{ads}} \, = \,\left( {1/55.5} \right)exp{\text{ }}\left( { - \Delta G^{ \circ } _{{ads}} /RT} \right)$$

**Figure 8:**
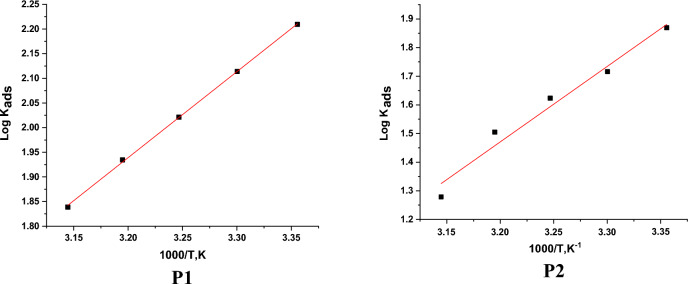
Log K_ads_ vs. T diagrams obtain from Langmuir.

In which, ΔG^o^_ads_ the regular free adsorbent, 55.5 ML^−1^ water dosage in solution. The data pattern revealed that the negative sign of ΔG^o^_ads_ was caused by the stability of the adsorbed porphyrin molecules and the spontaneity of the adsorbed on the SS304 surface^48^. Using the fundamental Van’t Hoff’s equation 7, a straight line emerges from drawing log K_ads_ vs. (1/T) (Fig. 8). ΔH°_ads_ were obtained from the slope:

7 $$log \, K_{ads} \, = \, - \Delta H^{o}_{ads} / \, 2.303RT\, + \,\Delta S^{o}_{ads} /R$$

By getting ΔH^o^_ads_ from the slope of Eq. (7), ΔS^o^_ads_ was designed using Eq. (8),

8 $$\Delta S^{o}_{ads} \, = \,\left( {\Delta H^{o}_{ads} - \Delta G^{o}_{ads} } \right)/ \, T$$

The determined values of adsorption parameters were introduced in Table 5. From Table 5, it was noticed that the K_ads_ data demonstrated that the adsorption coefficient reduces as temperature rises. Given that K_ads_ was higher at 298 K than it was at 318 K, it is likely that a greater amount of the derivative was adsorbed onto the surface of the SS304. This suggests that lower temperatures are advantageous for the inhibition process. “The negative ΔG°_ads_ values demonstrate both the spontaneity of the process. Values of ΔG^o^_ads_ lower than − 20 kJ mol^−1^ (18.4–22.5 kJ mol^−1^) are consistent with the electrostatic interaction between the charged molecules and the charged metal (physical adsorption)^49, 50^. The negative sign of ΔH°_ads_ indicates that the adsorption of derivative molecules is an exothermic, this means that the adsorption process may be physical or chemical, but because the values of ∆H^°^_ads_ are less than 80 kJ mol^−1^, so the investigated derivatives are physically adsorbed onto the SS304 surface”. The corrosion inhibitor is adsorbing to the surface of SS304, and the entropy of the system is decreasing, according to the negative data of the entropy change of adsorption ∆S^o^_ads_. This is attributable to the exothermic nature of the absorption manner, which can be seen by the negative sign ∆H^o^_ads_.

**Table 5:** Thermodynamic parameters obtain from Langmuir.

Inhibitor	Temp., K	R^2^	K_ads_ M^−1^	− ∆Gº_ads_ kJ mol^−1^	− ∆Hº_ads_ kJ mol^−1^	− ∆Sº_ads_ (J mol^−1^ K^−1^)
**P1**	298	0.9887	162	22.5	33	75.5
	303	0.9998	130	22.3		73.7
	308	0.9986	105	22.2		71.9
	313	0.9988	86	22.0		70.3
	318	0.9789	69	21.8		68.5
**P2**	298	0.9897	74	20.6	50	69.1
	303	0.9999	52	20.1		66.1
	308	0.9888	42	19.8		64.4
	313	0.9987	32	19.4		62.1
	318	0.9789	19	18.4		57.7

### Electrochemical measurements

#### Polarization (PDP) measurement

**Figure 9:**
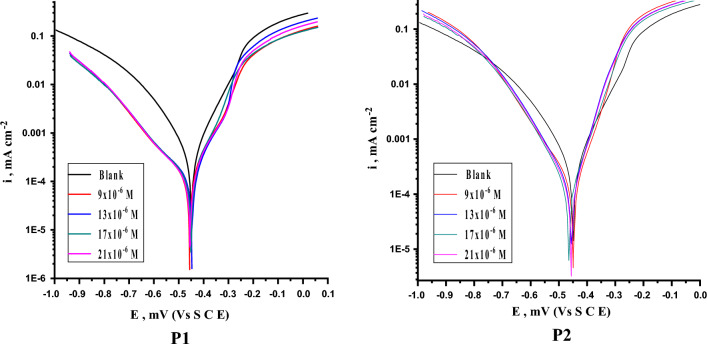
PDP plots for the dissolution of SS304 in 2 M HCl in the absence and attendance of altered dose of porphyrin derivatives.

PDP bends of SS304 in 2 M HCl attendance and lack of altered doses of the porphyrin derivatives (P1 & P2) are presented in Fig. 9. Table 6 lists the electrochemical characteristics that were derived by Tafel extrapolation at the corrosion potential (E_corr_), including current density (i_corr_), corrosion potential (E_corr_), anodic (β_a_) and cathodic (β_c_) slopes. When the inhibitor concentration is raised, it has been observed that i_corr_ decreases. The polarization curves were used to compute IE % and (θ) (Eqs. 9 & 10):

9 $$IE \, \% \, = \, 1 \, {-} \, \left( {i_{corr} / \, i^{o}_{corr} } \right)$$

10 $$\theta \, = \, IE\% \, /100$$

where, i^o^_corr_ and i_corr_ are the current corrosion densities existence and nonexistence of chemicals, respectively. For the test specimens in the 2 M HCl solution, the polarization resistance (R_p_) was designed using the Stern-Geary equation (11) below in both the existence and lack of porphyrin (P1 & P2) inhibitor^51^.

11 $$R_{p} = \beta _{a} \beta _{c} /{\text{ }}2.303i_{{corr}} (\beta _{a} + \beta _{c} )$$

**Table 6:** Parameters obtain from PDP technique.

Comp.	Conc., × 10^6^, M	− E_corr._, mV vs. SCE	i_corr_ μA cm^−2^	− β_c_, mV dec^−1^	β_a_, mV dec^−1^	R_p_ Ω	R_corr,_ mpy	θ	IE%
**P1**	Blank	498	443	135	97	131	187	–	–
	9	463	79	112	91	396	55	0.821	82.1
	13	455	67	105	86	467	44	0.848	84.8
	17	463	41	120	89	555	40	0.907	90.7
	21	458	33	117	79	539	38	0.925	92.5
**P2**	9	487	95	119	88	328	67	0.785	78.5
	13	485	81	125	97	448	53	0.817	81.7
	17	477	63	115	95	461	49	0.857	85.7
	21	483	51	122	90	536	42	0.885	88.5

It is obvious that the effectiveness of inhibition gradually increases as inhibitor dose increases, at 25 °C with additions of 21 × 10^–6^ M of the studied corrosion inhibitors P1 & P2, the highest inhibition effectiveness of 92.5% and 88.5% were achieved, respectively. i_corr_ significantly decreases as corrosion inhibitor dosage is increased, reaching a minimum data of less than 33 and 51 µA cm^−2^ at the same dosage of maximum efficiency for P1 & P2, respectively. Also, the polarization resistance (R_p_) increases from 131 to 536 Ω. “These findings confirm the high inhibition and film-forming ability of the investigated compounds^52^. According to the parallel cathodic Tafel lines, the addition of inhibitors to the 2 M HCl solution does not alter the hydrogen evolution mechanism or the reduction of H^+^ ions at the SS304 surface, which mostly happens through a charge transfer mechanism^53^. The inhibitor may be categorized as either anodic or cathodic depending on whether the change in corrosion free potential E_corr_ following the addition of the inhibitor is greater than 85 mV_SCE_ in either direction. Otherwise, it is thought that the inhibitor has an impact on both processes. The total change in E_corr_ in this investigation following the addition of corrosion inhibitor was found to be negligible, or less than 85 mV relative SCE in the direction of polarization (40, 21 mV for P1 & P2, respectively). This establishes that the inhibitor is a mixed-type inhibitor since it inhibits both the anodic and cathodic processes^54, 55^. The positive E_corr_ displacement indicates that the inhibitor is a mixed-inhibitor, but that the anodic reaction is predominate”. According to % IE values represented in Table 6, the inhibiting properties of the studied inhibitors at highest concentrations 21 × 10^–6^ M can be given in the following order: P1 > P2 with IE % values 91.7 and 89.5, respectively. These results are in good agreement with the results obtained from MR and EIS measurements.

#### Electrochemical impedance spectroscopy (EIS) tests

**Figure 10:**
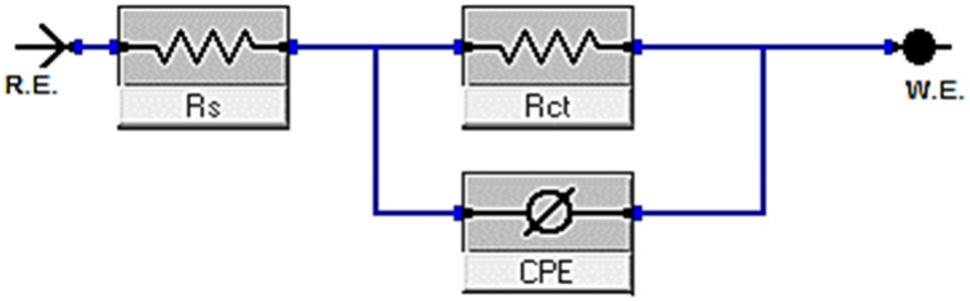
Fitting experimental EIS data with an equivalent circuit.

Another essential electrochemical method that is frequently used to research and give in-depth insight into utilize of corrosion hindrance to protector compared to corrosion is electrochemical impedance spectroscopy. The kinetic and mechanistic details of EIS systems are usefully provided^56^. The associated frequency is used to derive impedance data expressed in real *Z'* and imaginary *Z"* figures, which are then used to build a mathematical relationship shown as a Nyquist plot. A number of significant elements, including R_ct_, R_s_, and C_dl_, can be included in an equivalent circuit model of the system under study, which consists of a working electrode, an electrolytic solution, and an adsorbed inhibitor. The interpretation of analogous circuit components enables a reliable analysis of the corrosion protection process when utilizing corrosion inhibitors. R_ct_ has a direct relationship with the efficacy of corrosion inhibition, whereas C_dl_ lower with improved protection^57^. By contrasting impedance curves in the attendance and lack of the tested porphyrin inhibitors, the effectiveness of corrosion inhibition is examined. In order to assess the observed impedance of an electrolyte subject to uniform corrosion, Fig. 10 depicts a typical equivalent electrical circuit. As previously mentioned, the circuit consists of R_ct_, the electrochemical solution resistance R_s_, and the constant phase element (CPE), which is utilized to represent the non-ideal behavior of the double layer, which is mostly related to insufficient surface coverage and surface roughness. For modeling iron-acid interface corrosion previously, use the circuit below^58^.

**Figure 11:**
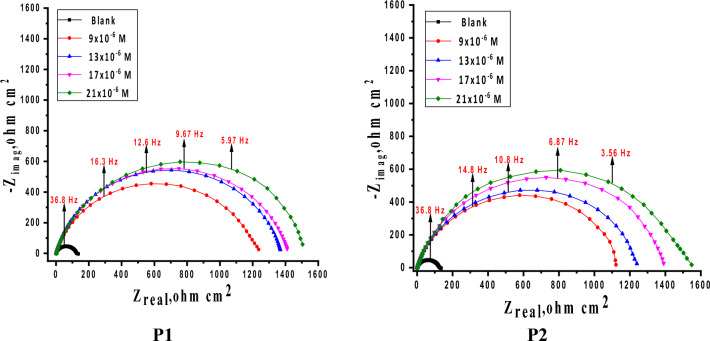
Nyquist plots for SS304 corrosion in the 2 M HCl solutions in the existence and nonexistence of various doses of porphyrin derivatives at 25 °C.

**Figure 12:**
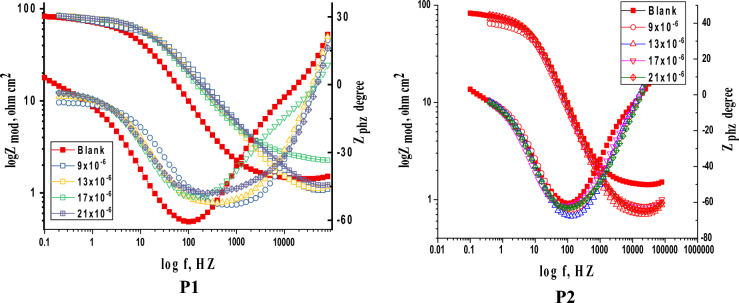
Bode plots for SS304 in 2 M HCl without/with altered dosages of porphyrin derivatives at 25 °C.

Figures 11, 12 display the Nyquist and Bode curves of SS304 in 2 M HCl solutions contain altered doses of porphyrin derivatives (P1 & P2) at 25 °C. These Nyquist diagrams in 2 M HCl are not perfect semicircles due to surface heterogeneities, roughness effects (Fig. 11), inhibitor adsorption, and deviations in the properties or compositions of layers surface^59, 60^, “which can be connected to the frequency dispersion effect. The curves described by a single capacitive semicircle, indicates that the corrosion process was mainly charged-transfer controlled”^61^. Equation (12) describes the impedance of a constant phase element (CPE):

12 $$Z_{CPE} = Y_{0}^{ - 1} \left( {j\omega } \right)^{ - n}$$

Where Y^0^ denotes the CPE's magnitude, the Y^o^ values which is attributed to the formation of double layer are smaller for inhibited solutions as compared to the uninhibited solution and n is the phase shift which measures the metal surface homogeneity and lies between 0 and 1. It is possible that P1 and P2 molecules interacted with the surface of electrode and protected the exposed electrode sites from damage. Using Eq. (13), the data of the CPE parameter Y^0^ and n can be used to compute the values of the interfacial capacitance C_dl_^62^:

13 $$C_{dl} = Y_{0} \left( {\omega_{max} } \right)^{{{\text{n}} - 1}}$$

Indeed, as “P1 & P2 concentration increased, R_p_ values raised while C_dl_ decreased, indicating that P1 & P2 was acting at the steel/acid interface. This is because inhibitor molecules replaced corrosive ions and water molecules on the substrate surface, increasing the thickness of the electric double layer and lowering the local dielectric constant^63^. The increases in the n value with the addition of P1 & P2 in 2 M HCl electrolyte (0.928–0.887) compared to that obtained in reference electrolyte (0.986) might be read as a certain reduction in the surface heterogeneity, but^64^. Bode plots in the absence and presences of porphyrin derivatives (P1 & P2) are given in Fig. 12. We may easily understand how the low frequency impedance modulus affects the inhibitory effect of porphyrins (P1 & P2) by observing this parameter. As seen in Fig. 12, the presence of P1 causes a greater rise in low frequency impedance modulus than the P2 solution does. This shows that P1 adsorption enhances SS304 corrosion resistance more than P2, and that the presence of porphyrin derivatives increases the low frequency impedance modulus relative to its absence. The single peak that was shown in the Bode plots for P1 and P2 demonstrated the existence of a single time constant, as indicated by the Nyquist plot. The equivalent circuit model simulation of Nyquist and Bode graphs demonstrates great agreement with experimental data. The evaluated values of Goodness of fit (χ^2^) (Table 7) support good quality of fitting and equivalent circuit used”. It is important to note that EIS studies support the superiority of P1’s protective capability over P2’s, which is compatible with the MR and PDP measurement findings. The data of the derived parameters of EIS fitting as C_dl_, R_ct_ and IE % are listed in Table 7.

**Table 7:** EIS parameters for SS304 corrosion in 2 M HCl in the absence and presence of various concentrations of porphyrin derivatives (P1 & P2) at 25 °C.

Comp.	Conc., M	n	Y_o,_ _(µ Ω_^−1^_ s_^n^_cm_^−2^_) × 10_^–6^	C_dl_ µF cm^−2^	R_ct_ Ω cm^2^	θ	IE%	Goodness of fit (χ^2^)
**P1**	Blank	0.986	335	320	124	–	–	18.56 × 10^–3^
	9 × 10^–6^	0.928	120	103	1237	0.899	89.9	20.88 × 10^–3^
	13 × 10^–6^	0.919	118	100	1373	0.909	90.9	22.78 × 10^–3^
	17 × 10^–6^	0.898	103	82	1417	0.912	91.2	18.10 × 10^–3^
	21 × 10^–6^	0.880	97	74	1546	0.919	91.9	16.27 × 10^–3^
**P2**	9 × 10^–6^	0.963	139	129	1031	0.879	87.9	18.54 × 10^–3^
	13 × 10^–6^	0.961	136	127	1212	0.897	89.7	21.15 × 10^–3^
	17 × 10^–6^	0.899	121	99	1375	0.909	90.9	18.87 × 10^–3^
	21 × 10^–6^	0.887	111	88	1461	0.915	91.5	17.57 × 10^–3^

## Surface analysis

### SEM tests

**Figure 13:**
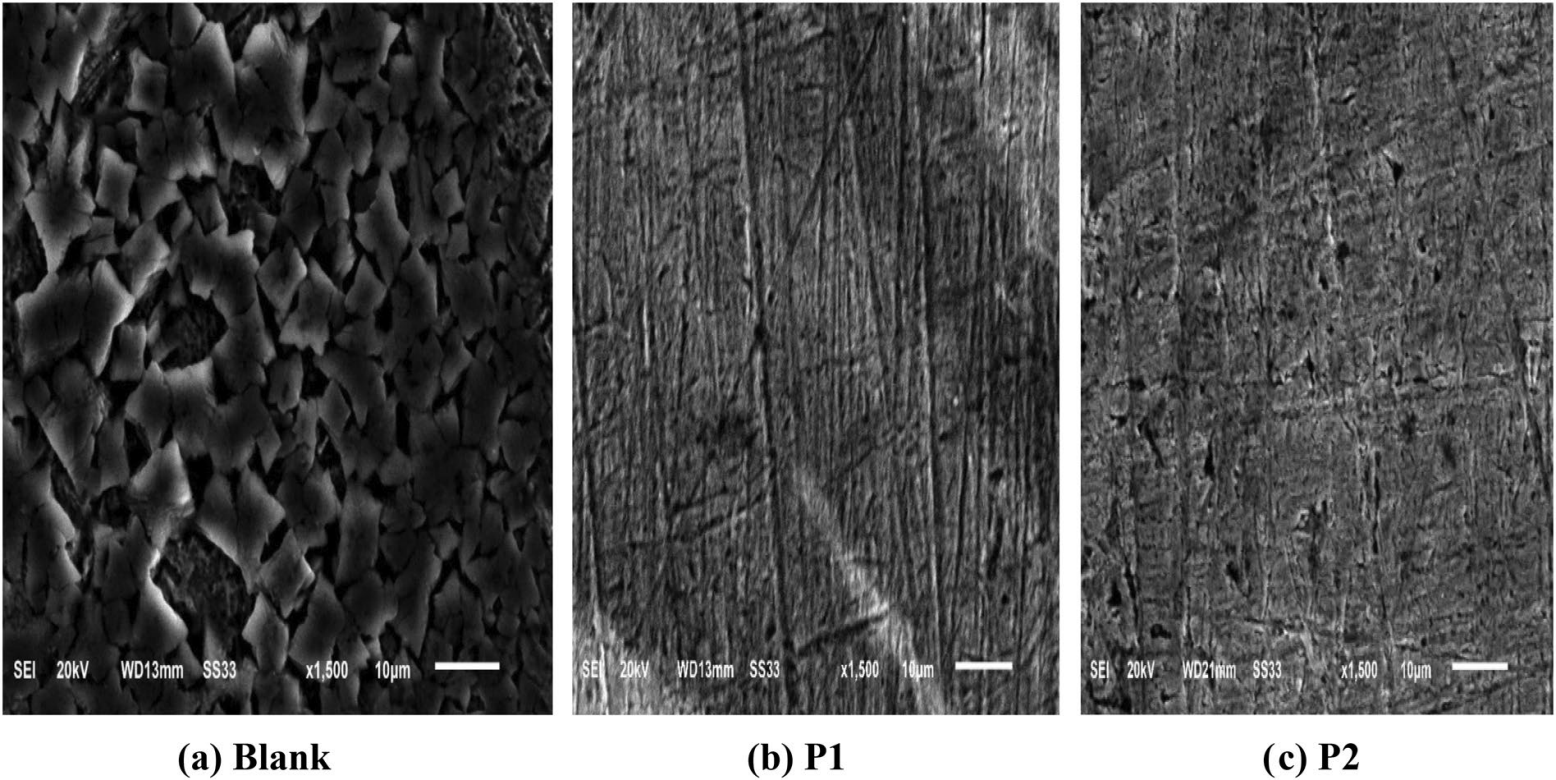
SEM pictures for (**a**) After of 24 h sinking in 2 molar HCl, (**b**, **c**) in the existence of porphyrin derivatives (P1 & P2).

The SS304 surface was examined using “SEM as depicted in Fig. 13 to see whether the surface morphology was altered by adding 21 × 10^–6^ M of porphyrin derivatives and without it. Following a 24-h dipping in HCl (2 M), the SS304 surface was analyzed using SEM, and the SS304 was tested both with and without the usage of 21 × 10^–6^ M from compounds P1 & P2. Without any inhibitor, corrosion in HCl (2 M) drastically weakened the surface of the SS304. The SS304 surface was observed after utilizing the inhibitors and found to be smooth. Because they provide a strong protective layer between the SS304 and the corrosive media, porphyrin derivatives (P1 & P2)” present in the solution reduce the rate of corrosion, which improves surface morphology and decreases surface roughness.

### AFM analysis

**Figure 14:**
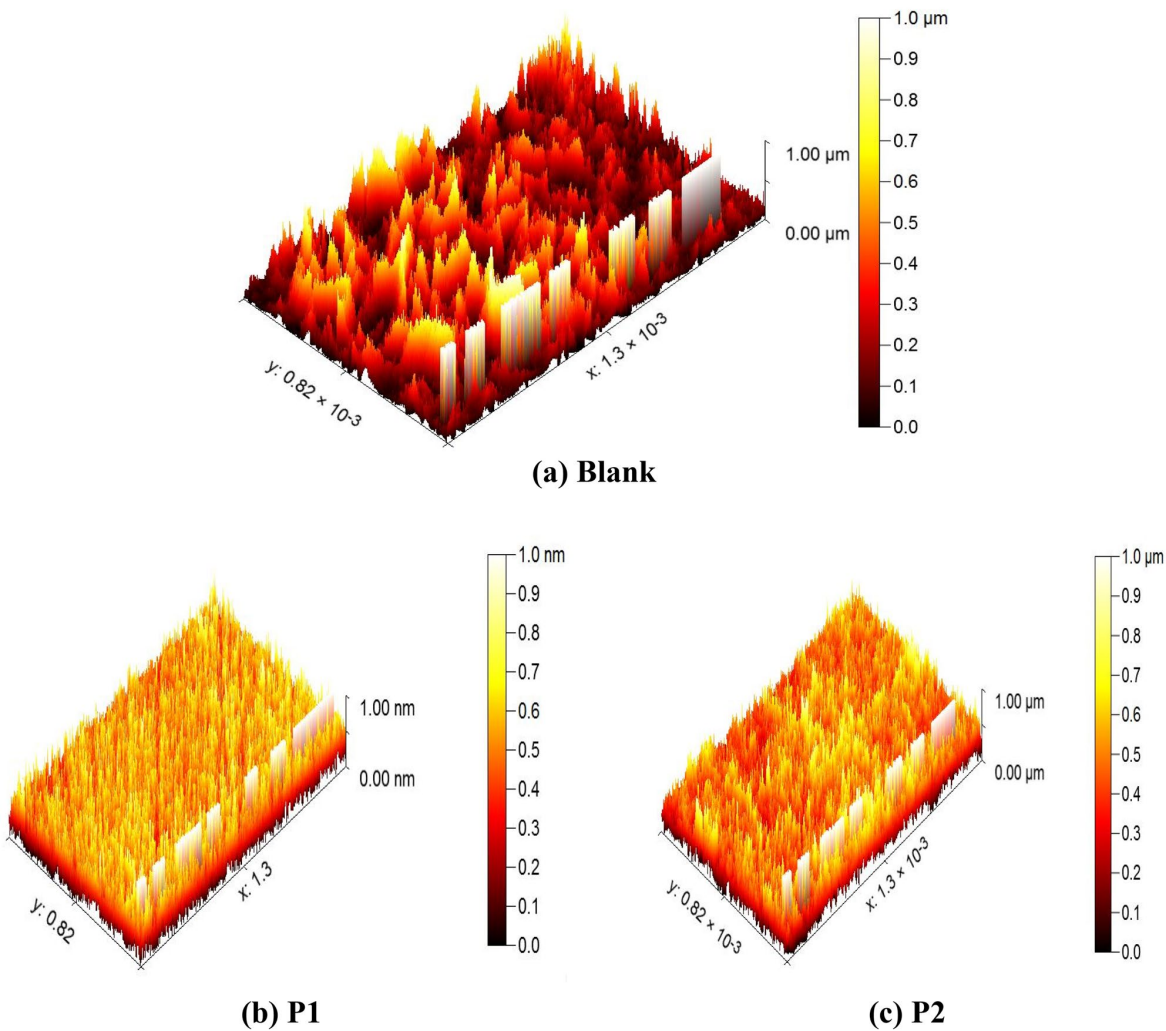
3D AFM morphology of SS304 surface in 2 M HCl (**a**), and in presence of 21 × 10^–6^ M P1 (**b**) & P2 (**c**), respectively.

According to the 3D image of the SS304 without the studied macrocyclic inhibitor, the metal surface has been repeatedly destroyed by the corrosive attacks of the 2 M HCl Fig. 14a. However, the 3D images (Fig. 14b, c) showing smoother surfaces than the blank demonstrate that the insertion of an inhibitor reduces corrosion of SS304 in the aggressive medium. The mean roughness (S_a_) of the films formed on the SS304 surface. The blank’s mean roughness are (820 nm) higher than those of the inhibitor it reduced to 147 nm and 190 nm Fig. 14b, c correspondingly in the presence of 21 × 10^–6^ M of porphyrin derivatives (P1& P2), under study and pure metal, proving the efficiency of the compound in protecting SS304 surface from corrosive medium.

## Computational methods

### Quantum chemical parameters

**Figure 15:**
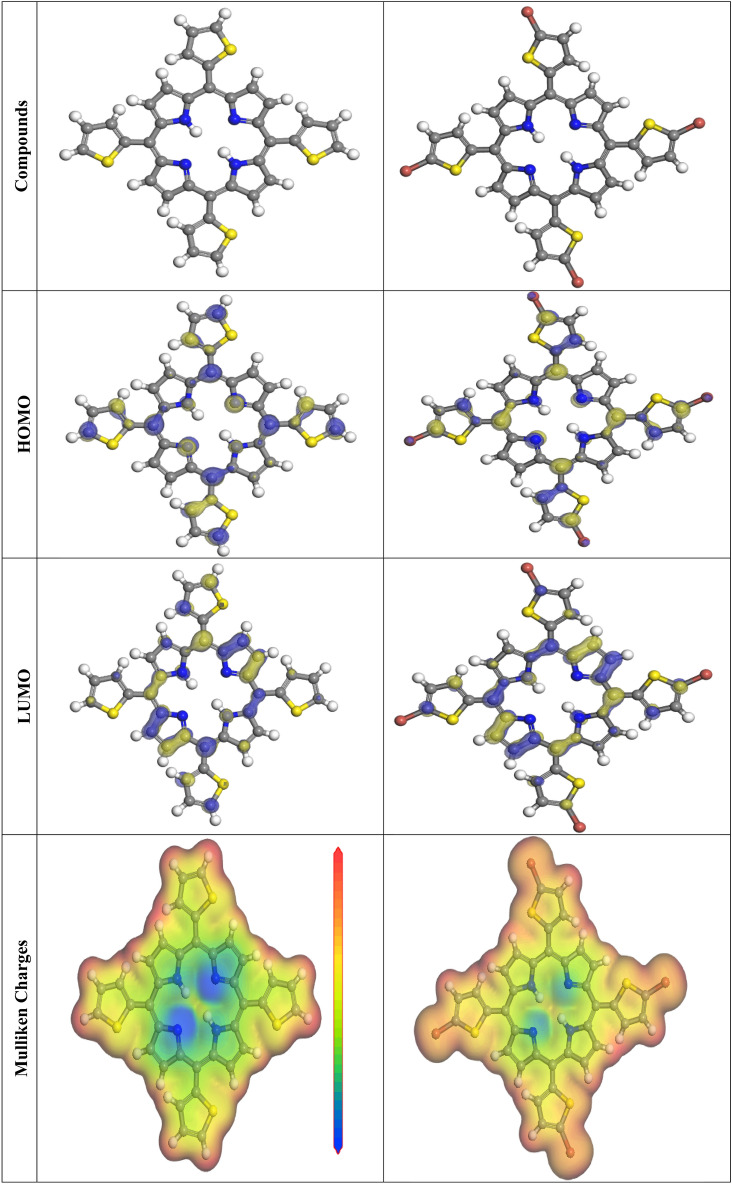
The optimized geometrical structure, (HOMO), and (LUMO) of the tested porphyrin derivatives (P1 & P2) at DMol3.

The lower energy band gap value, which is represented in the energy band gap ΔE_g_ (ΔE = E_HOMO_ E_LUMO_), indicates that organic molecules are highly reactive and exhibit excellent corrosion behaviour on the surface of SS304. An analysis of the impact of porphyrin derivatives (P1 & P2) molecule's orientation on inhibition performance was conducted using density function theory (DFT). As shown in Fig. 15, the optimized geometry, HOMO surface, and LUMO surface of studied inhibitors can be found. The parameters HOMO (E_H_), LUMO (E_L_), and dipole moment (μ) for porphyrin derivatives (P1 & P2) gradients were directly obtained from DFT (Table 8). “Eqs. (14–19) were used to calculate the energy gap (ΔE), electronegativity (χ), global hardness (η), global softness (σ), the fraction of electron transfer (ΔN) and back-donation (ΔE back-donation)”, was calculated as Koopmans’s theorem^65^ (depicted in Table 8) from the next balance:

14 $$\mu = - \chi = - \frac{{I_{p } + E_{A} }}{2}$$

15 $$\chi = \frac{{I_{p } + E_{A} }}{2}$$

16 $$\eta = \frac{{I_{P - } E_{A} }}{2}$$

17 $$\sigma = \frac{1}{\eta }$$

18 $$\omega = \frac{{\mu^{2} }}{2\eta }$$

19 $$\Delta E_{back donation} = - \frac{\eta }{4 }$$

**Table 8:** Quantum chemical data for the porphyrin derivatives (P1 & P2) under study.

Compound	P1	P2
*–E*_*HOMO*_*, eV*	4.533	4.492
*–E*_*LUMO*_*, eV*	3.743	3.666
*ΔE, eV*	0.790	0.830
*I*_*P*_*, eV*	4.533	4.492
*E*_*A*_*, eV*	3.743	3.666
*η, eV*	0.40	0.41
*σ, eV*	2.53	2.42
*ω*	21.67	20.14
*∆N*	3.62	3.54
*ΔE*_*back-donation*_	− 0.100	− 0.102
*Dipole moment (Debye)*	0.5932	1.6104

Numerous articles have discussed how higher values of E_HOMO_ and lower values of E_LUMO_ determine the greater electron-donating and accepting abilities of an inhibitor. Inhibitors are more reactive when a lesser value of ΔE is present. In this instance, the porphyrin derivatives (P1) ΔE value is lower in the gaseous phase while higher values for porphyrin derivatives (P2). In comparison to porphyrin derivatives (P1 & P2) molecules, these values suggest that the P1 molecule has a high degree of reactivity. Metals and inhibitors can be understood using the number/fraction of electron transfer (ΔN). If the ΔN value of an inhibitor is higher, it is found to have a stronger capability of donating electrons to metallic surfaces. Compared to porphyrin derivatives (P1 & P2) molecules, P1 exhibits greater amounts of ΔN in the gaseous phase, indicating that porphyrin derivatives (P1) exhibits a stronger inhibitory effect.

### Monte Carlo (MC) simulation

**Figure 16:**
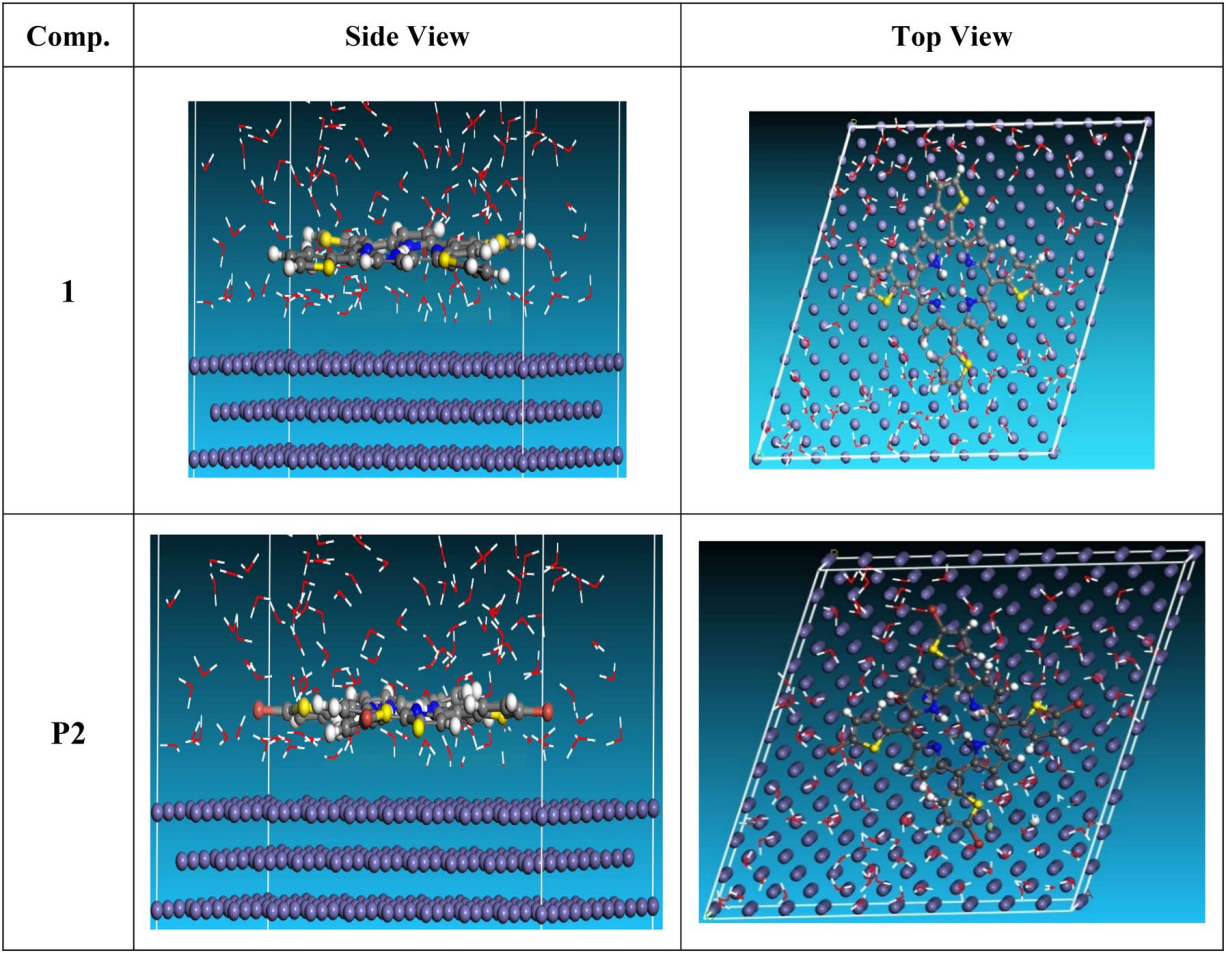
Adsorption configurations of the porphyrins molecules on the iron surface.

Monte Carlo simulation was utilized to find out more about the interactions between the molecules under study and the metal surface in an acidic and vacuum environment. Views of the more sturdy arrangement for the adsorption of porphyrin derivatives (P1 & P2) derivatives on the surface of cleaved Fe (1 1 0) from the top and sides (Fig. 16). MC stimulation done by adsorption lactor module detect the interaction between inhibitors and surface area of Fe (1 1 0) crystal with discovering the best adsorption sites^66^. Choosing the Fe (1 1 0) plane was based on its best stability and well-packed structure. Forcite module was used to optimize the geometry of porphyrin derivatives (P1 & P2). The Simulation annealing was used to calculate fine-quality adsorption using five cycles of 50,000 steps. This study investigates low-energy configurations of Fe (1 1 0)-inhibitor system in aqueous solution. In order to simulate corrosion in a real-life scenario, the simulation was conducted in an aqueous environment with water molecules. Table 9 presents the adsorption configuration which are nearly parallel in position resulting from relaxation of the inhibitor molecule on Fe (1 1 0). The descriptors computed from MC stimulation are in Table 9. The tabulated adsorption energies are − 4236.244 and − 3908.128 kcal mol^−1^ for porphyrins (P1), (P2) respectively. The outputs shows that the two inhibitors are efficient adsorptive inhibitors taking in respect that the better one is porphyrin (P1) which is attuned with the experimental results Rigid adsorption energies are − 4425.124 (P1) and − 4088.248 (P2) kcal mol^−1^ where porphyrin (P1) is the most negative, while for the deformation energies 188.88 (P1) and 180.12 (P2) kcal mol^−1^, also porphyrin (P1) is the highest value which confirm the greater inhibitory impact of porphyrin (P1) more than porphyrin (P2). dE_ad_/dN_i_ provide information about the metal adsorbents as if they are adsorbed or neglected, so when comparing dE_ad_/dN_i_ for inhibitors (− 253.19, − 234.80, − 307.35) kcal mol^−1^ and dE_ad_/dN_i_ for water (− 7.27, − 8.16, − 9.38) kcal mol^−1^, it’s found that the values in case of water is very low compared to that of the inhibitors proving the replacement of water molecules by inhibitor molecules. Based on theoretical modeling it’s obvious that porphyrins based proved to be powerful inhibitors for the carbon steel which is confirmed by experimental and spectral investigation. The prepared inhibitors are arranged P1 > P2 based on IE %.

**Table 9:** Monte Carlo simulation parameters of adsorption of porphyrins molecules on Fe (1 1 0) surface.

Structures	Adsorption energy	Rigid adsorption energy	Deformation energy	Compound dE_ad_/dNi	H_2_O dE_ad_/dNi
Fe (1 1 0)/Inhibitor P1/H_2_O	− 4236.244	− 4425.124	188.88	− 299.45	− 11.45
Fe (1 1 0)/Inhibitor P2/H_2_O	− 3908.128	− 4088.248	180.12	− 243.55	− 9.75

### Mechanism of inhibition

Porphyrin derivatives (P1 & P2) principally prevent SS304 corrosion by producing a dense barrier coating on the surface by transporting H_2_O molecules onto the surface and attaching to them^66^. It is found that the protective potential trends of the two Porphyrin derivatives be influenced by impact of substituent groups (Br) on the molecules' ability to give or take electrons. The IE % of inhibitors from all tested approaches rise in this order: P1 > P2. The greater effectiveness of the P1 inhibitor may be ascribed to the presence of N heteroatoms in its outer moiety, which are easily able to participate in surface-to-metal interaction and so efficiently reduce corrosion. However, because the inhibitor (P2) contains Br, which takes electrons, the protection's effectiveness is reduced, and the active site's electron density is also reduced. “Physical and chemical adsorption are two separate types. In contrast to the chemisorption process, which involves exchanging electrons or transporting them from the molecules to the iron's d-orbital in order to establish a coordination bond, the physisorption process necessitates the presence of both charged metal surfaces and charged molecules. Adsorption involving molecules and potential electrical density of energetic centers like N and S. In an acidic solution, the surface of the SS304 sample is positively charged^67^. The surface of carbon steel undergoes electrochemical reactions in corrosive medium where the chloride ions coming from HCl besides the water molecules cause the dissolution of metal surface making it positively charged. The negative-charged metal surface created by the deposited Cl^−^ ions on the SS304”. The cationic part of the Porphyrin molecules adsorbs on carbon steel surface forming a protective film against corrosive medium, this defined as physisorption.

## Conclusion

The main conclusions drawn from all these studies of macrocyclic compounds are:-

1. The IE % of all macrocyclic compounds improve with rise in inhibitor doses whereas it lower with the rises of temperature.
2. The IE % of all composites follows the order: P1 > P2 in 2 M HCl solution. This can be attributed to the altered in molecular structures and the type of the donating atoms.
3. The adsorption of all the composites on SS304 surface from the acidic solutions conforms Langmuir's isotherm.
4. P1 & P2 increase R_ct_ values and decrease i_corr_ values in 2 M HCl solution.
5. The EIS, PDP and MR tests are in good agreement.
6. The experimental finding agrees well with the theoretical calculations.
7. SEM and AFM investigation for SS304 surface revealed the presence of a protective film, which protect SS 304 alloy against the corrosive media.

### Supplementary Information

Supplementary Figures.

## Data Availability

All data generated or analyzed during this study are included in this published article and its supplementary information files.
