# Supplementary material for: Novel porphyrin derivatives as corrosion inhibitors for stainless steel 304 in acidic environment: synthesis, electrochemical and quantum calculation studies
Source: Sci Rep. 2023 Oct 16;13:17593. doi: 10.1038/s41598-023-44873-2 (PMC10579412; doi:10.1038/s41598-023-44873-2)
Supplement: Supplementary file 1 — Supplementary Figures. [file 41598_2023_44873_MOESM1_ESM.docx]

**
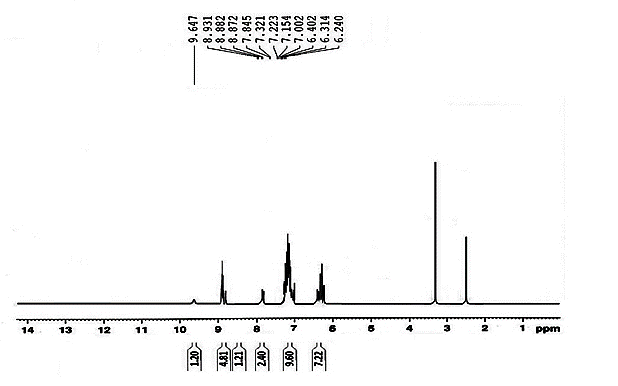
**

**Fig. S1 ^1^H-NMR spectrum of compound P1**

**
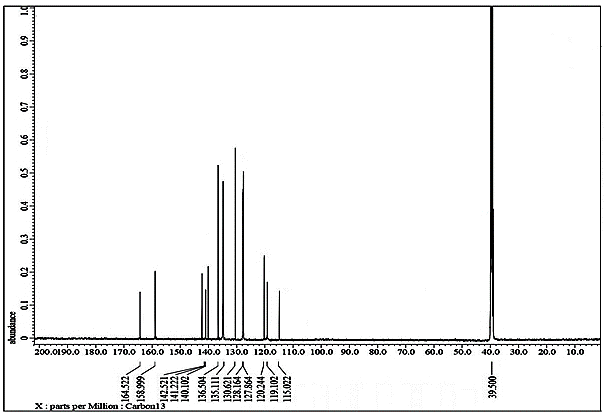
**

**Fig. S2 ^13^C-NMR spectrum of compound P1**

**
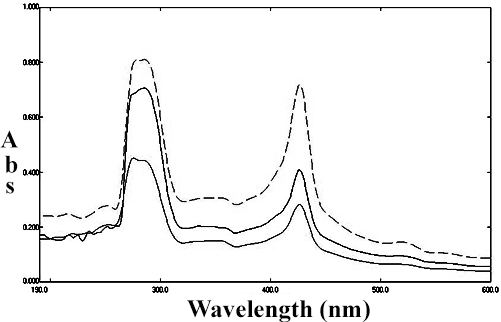
**

**Fig. S3 UV spectrum of compound P1**

**
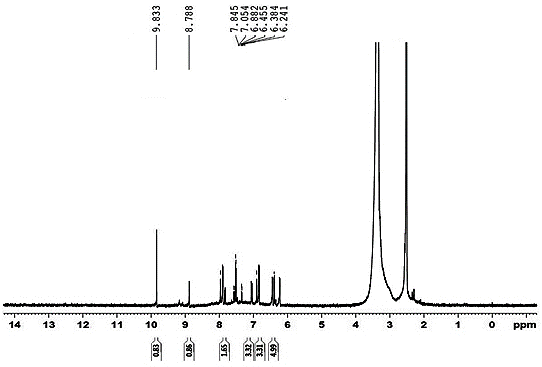
**

**Fig. S4 ^1^H-NMR spectrum of compound P2**

**
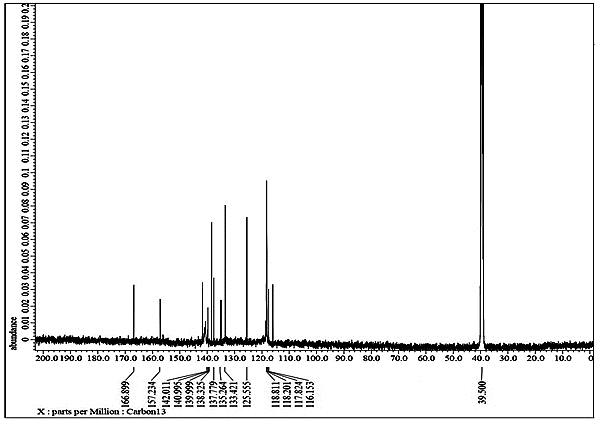
**

**Fig. S5 5 ^13^C-NMR spectrum of compound P2**


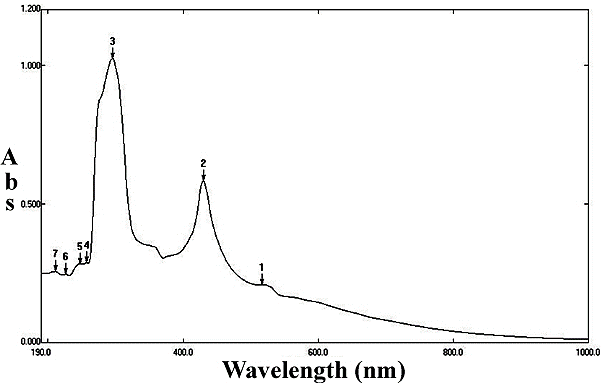


**Fig. S6 UV spectrum of compound P2**
